# Sleep Disturbances are a Significant Predictor of Chikungunya Arthritis Flare Severity

**DOI:** 10.33696/immunology.3.098

**Published:** 2021

**Authors:** Sarah R. Tritsch, Richard Amdur, Liliana Encinales, Andres Cadena, Paige Fierbaugh, Geraldine Avendaño, Carlos Andres Herrera Gomez, Karol Suchowiecki, Evelyn Mendoza-Torres, Wendy Rosales, Dennys Jimenez, Carlos Alberto Perez Hernandez, Alfonso Sucerquia Hernandez, Paula Bruges Silvera, Yerlenis Galvis Crespo, Alberto David Cabana Jimenez, Jennifer Carolina Martinez Zapata, Christopher N. Mores, Gary S. Firestein, Gary Simon, Aileen Y. Chang

**Affiliations:** 1 Milken Institute School of Public Health, The George Washington University, Washington, DC, USA; 2 Allied Research Society, LLC, Barranquilla, Colombia; 3 Clinica de La Costa Ltda., Barranquilla, Colombia; 4 School of Medicine and Health Sciences, The George Washington University, Washington, DC, USA; 5 Faculty of Health Sciences, Universidad Libre, Barranquilla, Colombia; 6 University of California, San Diego, USA

**Keywords:** Chikungunya, Arthritis, Pain, Inflammation, Sleep, Cytokines, T regulatory cells

## Abstract

**Objective:**

The primary objective of this research was to explore the link between sleep and flare pain associated with chikungunya virus (CHIKV) infection. The secondary objective was to investigate if cytokines and T regulatory (Treg) cells have an influence on this relationship.

**Methods:**

A cross-sectional study was performed using data collected in Barranquilla, Colombia, which enrolled patients with and without chronic arthritis with a history of chikungunya infection. Flare severity was measured by a version of the Outcome Measures in Rheumatoid Arthritis Clinical Trials (OMERACT) flare questionnaire adapted for CHIKV arthritis, including metrics for pain, difficulty with physical activity, fatigue, stiffness and difficulty maintaining social activities due to arthritis that contribute to flare severity. In addition, four sleep disturbance items, five inflammatory cytokine levels, four anti-inflammatory cytokine levels, and six Treg levels were measured. Then, multivariable linear regression models were used to test the direct and indirect effects of flare-pain on sleep disturbance, and to determine whether this relationship was mediated by cytokines or Tregs. Finally, the SAS CALIS procedure was used to test path models showing possible causal effects with mediators and confounds.

**Results:**

The analysis showed that sleep disturbance is positively correlated with CHIKV arthritis flare pain, and that it is a significant predictor of flare severity after adjusting for demographic variables, cytokine, and T cell levels. Further, neither T cells nor cytokines mediate the pain/sleep relationship in CHIKV arthritis.

**Conclusion:**

There is a strong association between sleep disturbance and arthritis flare pain and severity; however, this relationship is not mediated by cytokines or T cells. Since this study is unable to determine causation, further research is needed to determine the mechanism underlying the relationship between sleep disturbances and CHIKV arthritis flares.

## Introduction

Chikungunya virus (CHIKV) is an alphavirus transmitted by mosquitoes that causes debilitating arthritis. In a cohort of 500 Colombian patients, one quarter reported joint pain 20 months after infection, and further, 1 out of 8 had joint pain three years after infection [1]. For treatment of acute CHIKV symptoms, the CDC recommends acetaminophen and non-steroidal anti-inflammatory drugs (NSAIDs); however, there are currently no effective therapeutics or vaccines available [2].

There are many similarities between post-CHIKV arthritis and rheumatoid arthritis (RA), including a higher incidence in older patients and in women, as well as relapsing-remitting symptoms including joint pain, swelling, and morning stiffness [1]. While RA inflammation is due to an autoimmune response rather than viral infection, similarities in the cytokine secretion and induced genes in mouse models have been seen for both CHIKV infection and RA [3].

Previously, Chang et al. found that low levels of IL-2, IL-4, IL-13, and TNF-alpha during the acute phase of CHIKV infection was predictive of persistent joint pain [4]. In addition, Chow et al. found an increased level of IL-6 was associated with persistent joint pain, while Sepúlveda-Delgado et al. suggested IL-6 could be used to predict CHIKV-induced arthritis due to the strong relationship between IL-6 levels in the acute phase and severity of joint involvement and delay in musculoskeletal symptom resolution [5,6]. Furthermore, C-reactive protein (CRP), an inflammatory marker used in the diagnosis of RA, is also elevated during acute CHIKV infection [5].

Studies have shown that sleep impacts many aspects of the human body, including the immune system. While research investigating the relationship between sleep and CHIKV-associated arthritis is lacking, many studies have associated a lack of sleep or disturbances in sleep with increased disease activity and pain from RA [7-11]. Irwin et al. suggested that partial night sleep deprivation could activate pain, especially in populations with heightened signaling of neurobiological mechanisms, such as inflammation signaling in RA patients [10]. In fact, increases in inflammatory markers due to lack of sleep have even been described in healthy volunteers. Haack et al. described a significant increase in plasma IL-6 levels in healthy volunteers who had 4 hours of sleep versus 8 hours of sleep per night over 12 days. Furthermore, elevated IL-6 levels were significantly associated with increases in pain in response to less sleep, leading the authors to postulate that a lack of sleep could establish and maintain an association with pain and inflammation [12]. Immunity and sleep are influenced bi-directionally, and it is expected that sleep promotes the host’s immunity, affecting the outcome of an infection and even the uptake of vaccinations [7].

To better understand how sleep behaviors impact CHIKV patients with persistent joint pain after infection, a statistical analysis was performed using data collected during a case-control study in Barranquilla, Colombia. Our hypothesis was that there was a link between the development of CHIKV-associated persistent joint pain and the sleep habits of the patient. Secondarily, we also hypothesized there was an association between sleep habits and the cytokine profiles and T regulatory cell activity of patients, which may or may not lead to CHIKV-associated joint pain.

## Methods

### Ethics statement

This cross-sectional study (IRB no. 121611, Trans no. 28283) was approved by the ethics committee of the Clinica de La Costa Ltda. and the George Washington University Committee on Human Research. Written informed consent was obtained from all participants, and all samples were collected by qualified medical personnel.

### Participants and questionnaires

Patients with and without self-reported arthritis with a history of chikungunya infection were recruited from our chikungunya cohort in Colombia. Patients who reported no chronic arthritis symptoms were defined as CHIKV-controls. All patients signed informed consents. A survey and physical exam were conducted to ascertain demographic characteristics, disease activity, symptoms, arthritis flares, and quality of life.

Sleep variables were collected using the Patient-Reported Outcomes Measurement Information System (PROMIS)-29 questionnaire. These questions included the total number of hours of sleep per night and the time a person went to bed and awoke in the morning, as well as the four Likert scaled sleep items: “In the last 7 days the quality of my sleep was___;” “In the last 7 days my sleep was…restful,” “…I had trouble sleeping,” and “…I had trouble falling asleep.”

Flare pain was assessed on a continuous visual analog scale from 0-10. Flare severity was measured by a version of the Outcome Measures in Rheumatoid Arthritis Clinical Trials (OMERACT) flare questionnaire by Bartlett et al. adapted for CHIKV arthritis, including metrics for pain, difficulty with physical activity, fatigue, stiffness and difficulty maintaining social activities due to arthritis where each metric was scored from 0–10 resulting in a composite score of 0–50 for the five domains [13].

### Serum cytokine evaluation

Sera were collected from blood samples within 12 hours of collection and stored at −80°C until analysis. Evaluation of the serum cytokine concentrations was performed using the Milliplex MAP Kit High Sensitivity Magnetic Bead Panel on a MAGPIX powered by Luminex XMAP technology, including interleukin (IL)-10, IL-1β, IL-6, tumor necrosis factor (TNF)-α, IL-12 (p70), IL-13, IL-17, IL-2, IL-4, and IL-5.

### T cell evaluation

PBMCs were isolated from whole blood samples using Ficoll-Paque Plus separation medium within 12 hours of collection and stored at −80°C until analysis, or moved to liquid nitrogen within 3 months of isolation. PBMCs were thawed and stained for flow cytometry and were analyzed using a BD Celesta Cell Analyzer. FlowJo software was used to evaluate the T cell populations and to quantify the populations of T cell subsets, including T regulatory (CD3+CD4+CD25hi/int+FoxP3) and T effectors (CD3+CD4+ with differential expression of CD45RA, CD62L, CD95), as well as activation markers suggestive of transient immune activation (CTLA4, Helios, and HLA-DR). For statistical analysis, the percent of T regulatory cells (Tregs) out of live PBMCs was used, and the percent positive CTLA4 (CTLA4+ Tregs), Helios (Helios+ Tregs), and HLA-DR (HLA-DR+ Tregs) out of total Tregs was used. The T effector cells (Teff) were also calculated as the percent out of live PBMCs.

### Statistical analysis

For a measure of inflammation, we initially used separate inflammatory and anti-inflammatory cytokine variables, each of which was scored as the mean of the component cytokines. However, path models using these as two separate variables only fit the data marginally. When we combined them into a single inflammation scale, the fit was much better. Therefore, we only report the results using the single inflammation scale here.

We used the SAS CALIS procedure for testing path models showing possible causal effects with mediators and confounds. This is a structural equation modeling program similar to AMOS, LISREL, or MPLUS, which provides estimates for path coefficients in the model, along with model fit statistics [14]. These include standardized root mean square residual (SRMR, <0.10 is acceptable fit), root mean square error of approximation (RMSEA, <0.10 is acceptable), comparative fit index (CFI, >0.8 is acceptable), and the adjusted goodness of fit index (AGFI, ≥0.80 is acceptable), as well as probability of close fit (>.50 is good) [15-17]. Supplementary Table 1 serves as a reference for fit indices for path models. Models with different causal orders can be compared based on their fit indices, to determine which is a better fit to the data. The significance and strength of individual paths in the model tells us which variables may have causal impacts on other variables. We first tested models that included flare severity and sleep disturbance, with inflammatory markers as mediators. We then added T cell marker levels (Treg and Teff). Finally, we added confounders (gender, education, and age). At each step, the model parameters were adjusted until models with good fit were found. In each step, we tested models in which sleep disturbance predicted flare severity, as well as those in which flare severity predicted sleep disturbance. Standardized path coefficients were used in order to be able to compare the strengths of different paths in each model on the same scale.

## Results

### Demographic characteristics

Cases and controls were predominately adults (mean 48.6 ± 1.7 years old for cases, 48 ± 7.3 years old for controls) and a majority were female (87.1% of cases, 62.5% of controls). All patients were Mestizo, and a majority of cases had at least a high school education (65.3%), while only 37.5% of controls had the same level of education.

### Path models for examining causal effects, mediators, and confounds, in flare-sleep relationship

**Figure 1:**
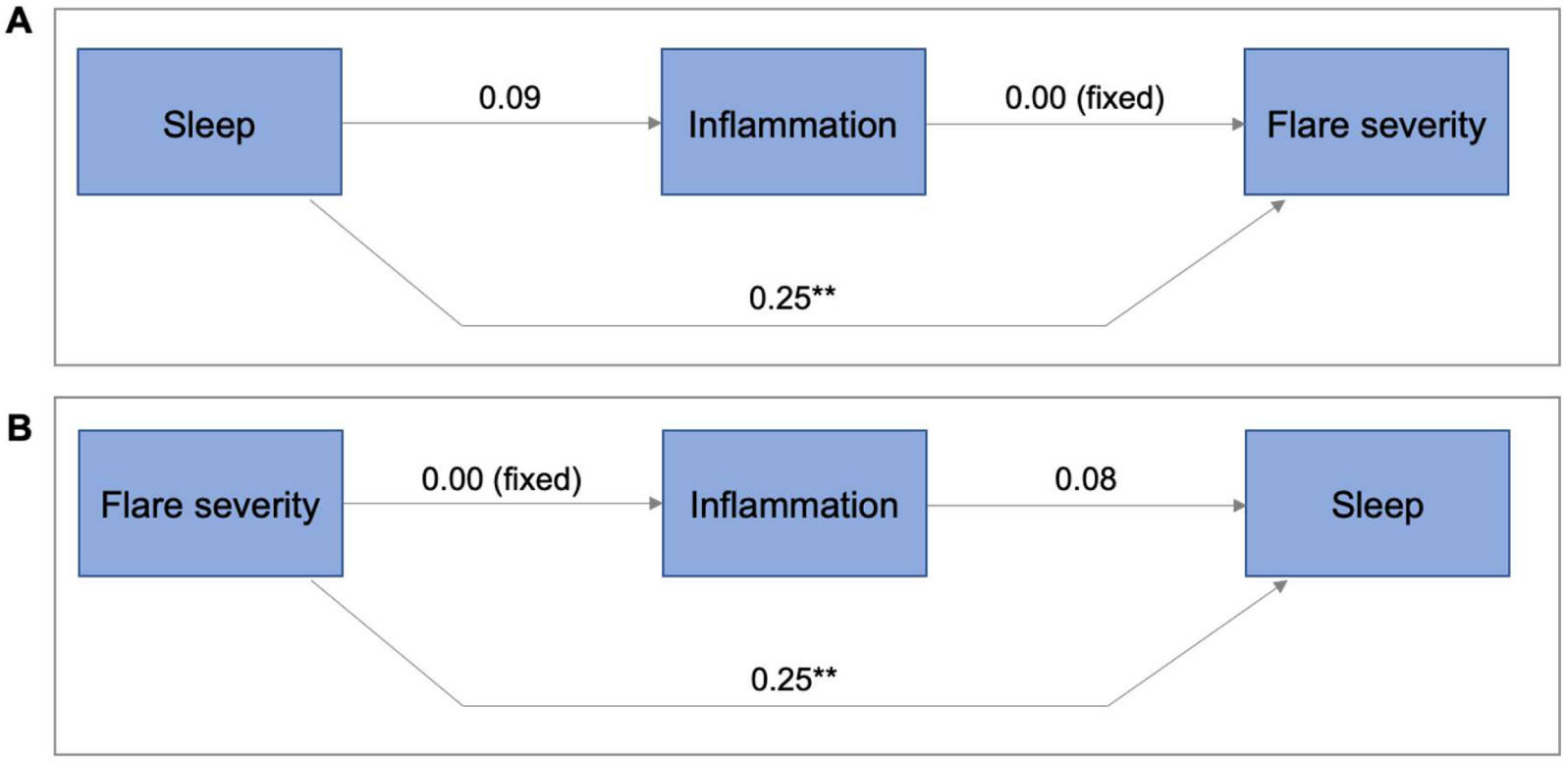
Path model showing inflammation as a mediator of the sleep-flare relationship. **A)** Sleep disturbance to flare severity (probability of close fit=0.98, AGFI 1.00, CFI 1.00, SRMR 0.00, RMSEA 0.00). There is a significant direct positive effect of sleep disturbance on flare severity. **B)** Flare severity to sleep disturbance severity (probability of close fit =0.85, AGFI=1.00, CFI=1.00, SRMR=0.01, RMSEA=0.00). Flare severity is a significant predictor of sleep disturbance. Neither relationships are mediated by inflammation. OMERACT = flare severity score; **p<0.01.

In the simplest path models, inflammation was included as a possible mediator of the path from either sleep disturbance to flare severity or from flare to sleep (Figure 1). The Spearman correlation matrix (Supplementary Table 2), shows the univariable associations between variables that were used in modeling. After adjusting for inflammation, both the path from sleep disturbance to flare severity and from flare severity to sleep disturbance remained significant. The sleep disturbance to flare severity model (Figure 1A) was a near perfect fit (probability of close fit=0.98, AGFI 1.00, CFI 1.00, SRMR 0.00, RMSEA 0.00). There is a significant direct positive effect of sleep disturbance on flare severity (*p*<0.01). For each 1 SD increase in sleep disturbance, the flare severity increased by 0.25 SD units. Inflammation was weakly affected by sleep disturbance, but has no effect on flare severity, so it did not act as a mediator here. The flare severity to sleep disturbance model (Figure 1B) fit was very strong (probability of close fit=0.85, AGFI=1.00, CFI=1.00, SRMR=0.01, RMSEA=0.00). Flare severity was a significant predictor of sleep disturbance (*p*<0.01), which was not mediated by inflammation.

**Figure 2:**
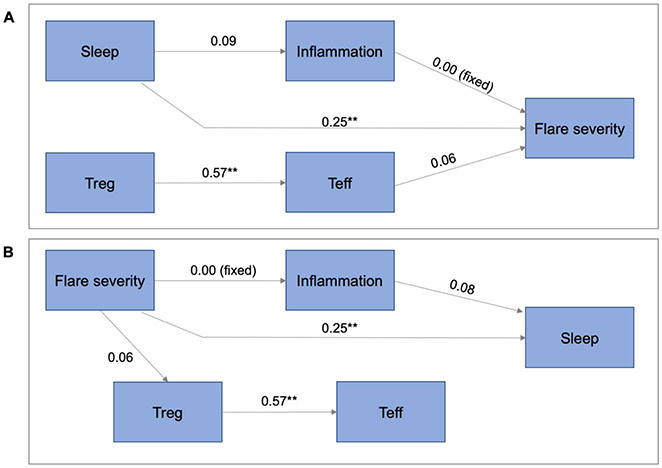
Path models in which inflammation and T-cells are potential mediators of the sleep-flare relationship. **A)** Sleep to flare (probability of close fit=0.59, AGFI=0.95, CFI=1.00, SRMR=0.05, RMSEA=0.00). Sleep disturbance has a significant direct positive effect on flare severity and is not mediated by inflammation or T-cells. There is no effect of sleep disturbance on T-cells. **B)** Flare to sleep (probability of close fit=0.71, AGFI=0.96, CFI=1.00, SRMR=0.05, RMSEA=0.00). The direct effect of flare to sleep is significant. There is no effect of flare severity on inflammation and only a weak effect on T-cells. Inflammation has a weak, non-significant effect on sleep disturbance. OMERACT = flare severity score; **p<0.01.

T-cells were then added to the previous model (Figure 2). Here, we again found that the direct path from sleep disturbance to flare severity, as well the direct path from flare severity to sleep disturbance remained significant, without being mediated by inflammation or T-cells. The sleep to flare model (Figure 2A) fit was good (probability of close fit=0.59, AGFI=0.95, CFI=1.00, SRMR=0.05, RMSEA=0.00). Sleep disturbance had a significant direct positive effect on flare severity, which remained as strong as in the previous model, and was not mediated by inflammation or T-cells. There was no effect of sleep disturbance on T-cells. The flare to sleep model (Figure 2B) fit was also good (probability of close fit=0.71, AGFI=0.96, CFI=1.00, SRMR=0.05, RMSEA=0.00). The direct effect of flare to sleep remained significant and the effect size remained the same as in previous models. There was no effect of flare severity on inflammation, and there was only a weak effect on T-cells. Inflammation had a weak, non-significant effect on sleep disturbance. Finally, when we added confounds to these models, in both the flare to sleep model, and the sleep to flare model, the direct paths remained significant, and there was no mediation by inflammation or T-cells.

**Figure 3:**
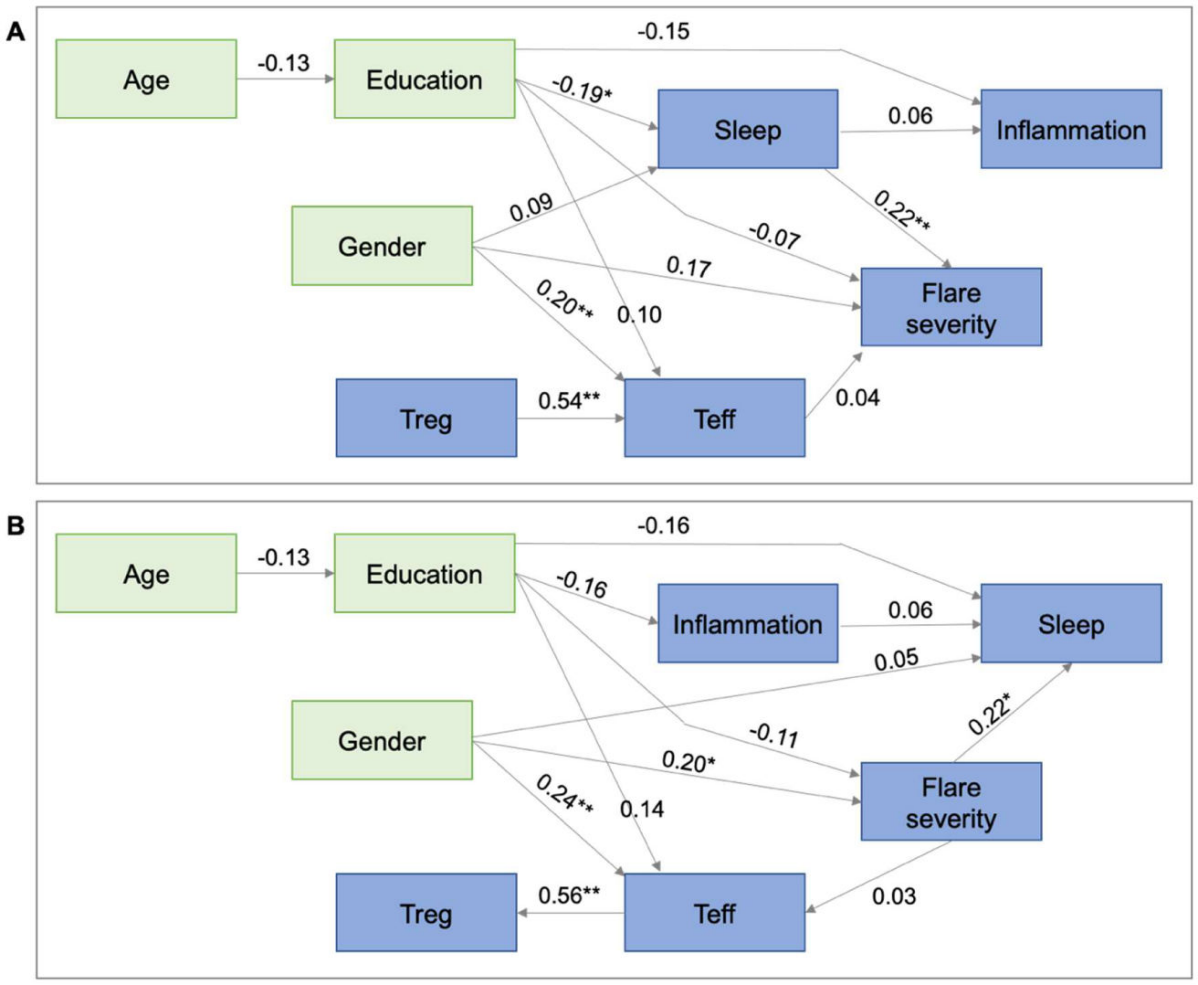
Final standardized path model including age, education, and gender as confounds, with T cells and inflammation as potential mediators. **A)** Sleep to flare model (probability of close fit=0.86, AGFI=0.94, SRMR=0.04, CFI=1.00, RMSEA=0.00). Sleep is a significant predictor of flare severity, after adjusting for confounds and possible mediators (0.01<*p*<0.05). Neither Tcells nor inflammation mediate this relationship. **B)** Flare to sleep model (probability of close fit=0.63, AGFI=0.92, SRMR=0.06, CFI=0.98, RMSEA=0.03). Flare severity has a significant effect on degree of sleep disturbance (0.01<*p*<0.05). This relationship is not mediated by inflammation. OMERACT = flare severity score; *p<0.05, **p<0.01.

Comparing the final sleep to flare vs. flare to sleep path models after adjusting for all confounds and mediators in the study (Figure 3) showed that the sleep to flare model fit the data better, with a *p* close fit of 0.86 vs. 0.63, while all the other fit statistics were also slightly better. The sleep to flare model (Figure 3A) fit was excellent (probability of close fit=0.86, AGFI=0.94, SRMR=0.04, CFI=1.00, RMSEA=0.00). Sleep was a significant predictor of flare severity, after adjusting for confounds and possible mediators (0.01<*p*<0.05). There was a 0.22 SD increase in flare severity for each 1 SD increase in sleep disturbance. Neither T cells nor inflammation mediated this relationship (i.e., there were no paths from sleep to Treg or Teff; there was a non-significant path from sleep to inflammation, but no path from inflammation to flare). Higher levels of education were associated with reduced sleep severity, but education had a non-significant effect on flare severity, so it is only a weak confounder. Age reduced education significantly, but had no direct effects on sleep or flare severity, while gender had non-significant effects on sleep and flare severity, so it was also a weak confound. The flare to sleep model (Figure 3B) fit was also very good (probability of close fit=0.63, AGFI=0.92, SRMR=0.06, CFI=0.98, RMSEA=0.03). Flare severity had a significant effect on degree of sleep disturbance (0.01<*p*<0.05). For each 1 SD increase in flare severity, there was a 0.22 SD increase in sleep disturbance score. This relationship was not mediated by inflammation, since there was no path from flare severity to inflammation, and inflammation had only a weak and non-significant effect on sleep disturbance. Education had direct negative effects on both flare severity and sleep disturbance, but neither was significant. Female gender was significantly associated with flare severity (being female was associated with a 0.2 SD increase in flare severity, 0.01<*p*<0.05) and with Teff level (females had a 0.24 SD higher level of Teff than males, 0.01<*p*<0.05). Teff level strongly predicted Treg level, but these did not act as mediators in the flare-to-sleep relationship, since neither had an impact on sleep, and Teff was only weakly influenced by flare severity. Age affected education but had no effect on other variables in the model.

These models suggest that further study of the effects of disturbed sleep is warranted in this patient population. The data also contradicts the hypothesis that the sleep-flare relationship is mediated by inflammation or T-cell activation.

## Discussion

Our key findings are that sleep disturbance is positively correlated with CHIKV arthritis flare pain and is a significant independent predictor of flare severity; however, there was no evidence that either flare pain or sleep disturbance directly affect or were affected by inflammatory cytokines, anti-inflammatory cytokines, or T cells. Specifically, we found a direct relationship between sleep and flare severity as measured by the OMERACT flare score adapted for CHIKV arthritis. Our findings linking sleep disturbances and arthritis flare pain are in accordance with previous studies of other types of arthritis [8,10].

Our analysis indicated that sleep was not directly related to inflammatory cytokines or T-cells responses. Instead, the results suggest that CHIKV arthritis patients were not sleeping well, and that this disrupted sleep or lack of sleep may contribute to arthritis flare severity. Other studies have noted similar findings and suggested alternative explanations for a lack of sleep in arthritis patients. Goes et al. suggested that pain, not inflammation, was the source of the association between sleep disturbance and RA [18]. Other studies have also shown that pain intensity is not always in proportion to levels of inflammation. A study by Lee et al. showed that RA patients had an impaired conditioned pain modulation (CPM), and thus a lower tolerance to pain, compared to healthy controls, and that the relationship between CPM and RA was confounded or mediated by sleep [19]. Further, depression can cause sleep loss in healthy individuals, and insomnia is a symptom of depression. Yilmaz et al. found that psychological stress and mood disorders, such as depression and anxiety, were independent factors for RA relapse periods, while Goes et al. found that depression was independently associated with sleep quality in RA patients [18,20]. Taken together with our findings, it suggests that depression, pain perception, discomfort, or other mood states associated with chronic pain could contribute to a lack of sleep, which could in turn cause increased flare-pain. Lee et al. also suggests that improvements in sleep and managing pain catastrophizing could prevent or help to reverse abnormal CPM [19].

Estrogens play a complex role in inflammation, creating a heightened inflammatory response in women compared to men, and putting women at greater risk of autoimmune diseases like RA [21]. In addition, a systematic analysis of pain experience in inflammatory arthritis showed that women report significantly more pain than men and persistently have worse pain scores through the course of the disease [22]. This could explain our findings that females reported greater flare severity than males.

There were multiple limitations to this study. First, while there is a strong association between sleep disturbance and flare severity, this study is unable to determine causation. Additionally, it is unclear from the questionnaire responses whether patients were getting less sleep due to an inability to sleep (i.e., from restlessness or joint pain), or if the limited amount of sleep was due to lifestyle choices. Lastly, our study involved flare severity, flare pain and sleep scores that were based on self-reported answers to questionnaires. These answers rely on participant recall and perceptions, which can be biased or incorrect. Future studies identifying barriers to adequate sleep in CHIKV arthritis patients may be of value. That is why it is important to use validated self-report scales, as we did here.

Further studies are required to understand the link between sleep and pain associated with post-CHIKV arthritis flares; however, this study suggests a relationship between pain levels and a lack of sleep or low-quality sleep. Thus, patients suffering from persistent post-CHIKV arthritis may benefit from addressing barriers to sleep.

## Supplementary Material

JCI-20-099_Supplementary file
