# Supplementary material for: Sleep Disturbances are a Significant Predictor of Chikungunya Arthritis Flare Severity
Source: J Cell Immunol. Author manuscript; Available in PMC 2021 Jul 27. (PMC8315573; doi:10.33696/immunology.3.098)
Supplement: JCI-20-099_Supplementary file [file NIHMS1724066-supplement-JCI-20-099_Supplementary_file.pdf]

Supplementary Table 1: Fit indices for path models.

MEASURE	Range	Indicator of good fit
CFI	0 to 1, 1 is perfect	>0.9 is good, >0.8 is marginal
RMSEA	0 to ∞, 0 is perfect	≤0.5 is good, ≤0.8 is reasonable, ≥0.10 is poor
AGFI	0 to 1, similar to R^2	≥0.90 is good, ≥0.80 is reasonable
SRMR	0 to ∞, 0 is perfect	<0.05 good, <0.10 acceptable

Supplementary Table 2. Spearman correlation matrix, showing the univariable associations between variables that were used in modeling.

Spearman Correlation Coefficients
Prob > |r| under H_0: Rho=0
Number of Observations

	Flare	Sleep	Inflamm-ation	Teff	Treg	Age	Gender	Education
**Flare**	1.00000<br><br>113	0.20392<br>0.0303<br>113	0.04204<br>0.6584<br>113	0.06634<br>0.4851<br>113	0.05110<br>0.5909<br>113	0.07675<br>0.4191<br>113	0.18000<br>0.0564<br>113	-0.11322<br>0.2346<br>112
**Sleep**	0.20392<br>0.0303<br>113	1.00000<br><br>113	0.11539<br>0.2236<br>113	0.01082<br>0.9095<br>113	0.00452<br>0.9621<br>113	0.11410<br>0.2288<br>113	0.08923<br>0.3473<br>113	-0.21012<br>0.0262<br>112
**Inflammation**	0.04204<br>0.6584<br>113	0.11539<br>0.2236<br>113	1.00000<br><br>113	0.20320<br>0.0309<br>113	0.10900<br>0.2505<br>113	0.00750<br>0.9372<br>113	-0.02808<br>0.7678<br>113	-0.20149<br>0.0331<br>112
**Teff**	0.06634<br>0.4851<br>113	0.01082<br>0.9095<br>113	0.20320<br>0.0309<br>113	1.00000<br><br>113	0.70936<br><.0001<br>113	0.14697<br>0.1203<br>113	0.22270<br>0.0177<br>113	0.09829<br>0.3025<br>112
**Treg**	0.05110<br>0.5909<br>113	0.00452<br>0.9621<br>113	0.10900<br>0.2505<br>113	0.70936<br><.0001<br>113	1.00000<br><br>113	0.15523<br>0.1006<br>113	0.07399<br>0.4361<br>113	0.06609<br>0.4887<br>112
**Age**	0.07675<br>0.4191<br>113	0.11410<br>0.2288<br>113	0.00750<br>0.9372<br>113	0.14697<br>0.1203<br>113	0.15523<br>0.1006<br>113	1.00000<br><br>113	0.11067<br>0.2432<br>113	-0.27019<br>0.0040<br>112
**Gender**	0.18000<br>0.0564<br>113	0.08923<br>0.3473<br>113	-0.02808<br>0.7678<br>113	0.22270<br>0.0177<br>113	0.07399<br>0.4361<br>113	0.11067<br>0.2432<br>113	1.00000<br><br>113	-0.06015<br>0.5287<br>112
**Education**	-0.11322<br>0.2346<br>112	-0.21012<br>0.0262<br>112	-0.20149<br>0.0331<br>112	0.09829<br>0.3025<br>112	0.06609<br>0.4887<br>112	-0.27019<br>0.0040<br>112	-0.06015<br>0.5287<br>112	1.00000<br><br>112
